# Association between the aromatase (*CYP19A1*) gene variant rs10046 and cardiovascular risk in postmenopausal women

**DOI:** 10.20945/2359-4292-2024-0087

**Published:** 2024-11-06

**Authors:** Betânia Rodrigues dos Santos, Gislaine Casanova, Thais Rasia da Silva, Karen Oppermann, Poli Mara Spritzer

**Affiliations:** 1 Unidade de Endocrinologia Ginecológica Hospital de Clínicas de Porto Alegre Divisão de Endocrinologia Porto Alegre RS Brasil Unidade de Endocrinologia Ginecológica, Divisão de Endocrinologia, Hospital de Clínicas de Porto Alegre, Porto Alegre, RS, Brasil; 2 Universidade Federal do Rio Grande do Sul Laboratório de Endocrinologia Molecular Departamento de Fisiologia Porto Alegre RS Brasil Laboratório de Endocrinologia Molecular, Departamento de Fisiologia, Universidade Federal do Rio Grande do Sul, Porto Alegre, RS, Brasil; 3 Hospital de Clínicas de Porto Alegre Divisão de Ginecologia e Obstetrícia Porto Alegre RS Brasil Divisão de Ginecologia e Obstetrícia, Hospital de Clínicas de Porto Alegre, Porto Alegre, RS, Brasil; 4 Universidade de Passo Fundo e Hospital São Vicente de Paulo Faculdade de Medicina Passo Fundo RS Brasil Faculdade de Medicina, Universidade de Passo Fundo e Hospital São Vicente de Paulo, Passo Fundo, RS, Brasil

**Keywords:** Aromatase gene variants, estradiol, ASCVD, menopause

## Abstract

**Objective:**

To assess the genotypic and allelic distribution of the rs10046 polymorphism in the *CYP19A1* gene and evaluate whether this aromatase gene variant is associated with cardiovascular risk in postmenopausal women.

**Materials and methods:**

This cross-sectional study analyzed repository-stored samples from 370 postmenopausal women aged 44-72 years. Clinical, metabolic, and hormonal data were collected. The patients’ estimated 10-year atherosclerotic cardiovascular disease (ASCVD) risk was calculated using the ASCVD Risk Estimator Plus, as recommended by the American College of Cardiology/American Heart Association. Genotyping of the rs10046 polymorphism of the *CYP19A1* gene was carried out using real-time polymerase chain reaction with allelic discrimination assays.

**Results:**

The participants had a mean age of 56.07 ± 5.58 years and a mean body mass index (BMI) of 27.73 ± 5.41 kg/m². The 10-year ASCVD risk was estimated to be low, borderline, intermediate, and high in 64.7%, 12.8%, 19.8%, and 2.7% of the participants, respectively. The CC genotype of the rs10046 polymorphism was associated with low estradiol levels (p = 0.003) and high ASCVD scores (p = 0.014). In a multivariate model, age (p < 0.001) and CC genotype (p = 0.021) were independently associated with higher ASCVD risk.

**Conclusion:**

The present study found that the CC genotype of the rs10046 polymorphism in the *CYP19A1* gene is associated with low estradiol levels and an increased ASCVD risk. Additionally, the results indicated that, among postmenopausal women, age and the CC genotype of rs10046 were associated with a high prevalence of ASCVD risk, independent of BMI.

## INTRODUCTION

Cardiovascular diseases (CVDs) are the leading causes of premature mortality. A recent review reported an estimated 18.6 million CVD-related deaths globally in the year 2019 (1). Recent integrated data from cohorts worldwide, including studies conducted by our group, have shown that 57.2% of incident CVD cases and 22.2% of deaths in women may be attributed to modifiable risk factors (2). Thus, identifying women's cardiovascular risk profiles is important to determine those who are most likely to benefit from intensive lifestyle modification alone, or from the addition of drug therapy to nonpharmacological measures. There are currently several tools available for estimating cardiovascular risk. One of the most widely used today is the atherosclerotic cardiovascular disease (ASCVD) Risk Estimator Plus, which is recommended by the American College of Cardiology and the American Heart Association (ACC/AHA) (3,4).

The postmenopausal period is characterized by a loss of ovarian follicular function, resulting in several changes related to decreased estrogen levels (5). Thus, postmenopausal women may present an unfavorable cardiovascular profile, linked to aging, decreased estrogen secretion by the ovaries, or both, and expressed by an adverse lipid profile, increased risk of insulin resistance, and high blood pressure, collectively contributing to increased CVD risk (3,6).

Estrogens are synthesized through the conversion of androgens by aromatase (CYP19A1), which is a cytochrome p450 enzyme encoded by the *CYP19A1* gene. Notably, CYP19A1 is abundantly expressed in several tissues, including the gonads, brain, and adipose tissue (7,8). Circulating hormones, local factors, transcription factors, and exogenous substances can influence aromatase activity and affect serum estradiol levels (8,9).

The *CYP19A1* gene, located on chromosome 15q21.2, comprises 10 exons, with exons 2 to 10 being transcribed and translated to produce the aromatase enzyme (10). The rs10046 polymorphism is an intronic single nucleotide polymorphism (SNP) located in the 3’ untranscribed region (3’UTR) of the *CYP19A1* gene. It involves the substitution of a cytosine with a thymine (C→T) and has been recognized for its involvement in modulating gene expression. While some studies show its association with estradiol levels (11-14), data concerning its relationship with cardiovascular risk in postmenopausal women are limited. Indeed, some studies have reported that the rs10046 polymorphism has been associated with coronary artery disease (15), hypertension (16), high triglyceride (17) and insulin levels, and the homeostatic model assessment of insulin resistance (HOMA-IR) index (18).

Based on these considerations, the aims of the present study were to assess the genotypic and allelic distribution of the rs10046 polymorphism in the *CYP19A1* gene and evaluate whether this aromatase gene variant is associated with cardiovascular risk in postmenopausal women.

## MATERIALS AND METHODS

### Study design and participants

In this cross-sectional study, we analyzed repository-stored samples from 370 postmenopausal women aged 44-72 years, without a clinical history of CVD and living in southern Brazil. The participants were prospectively recruited at the time they took part in previous studies conducted at our research center between 2005 and 2012 (19-21). The postmenopausal status was defined as a period of amenorrhea ≥ 12 months and/or amenorrhea ≥ 6 months plus follicle-stimulating hormone (FSH) levels ≥ 35 mIU/mL occurring after the age of 40 years. Women who had undergone hysterectomy with or without bilateral oophorectomy were excluded.

The serum samples were previously collected and stored in aliquots at −80 °C for use in laboratory tests. Additional blood samples were collected into EDTA tubes or spotted on FTA Elute cards (GE Healthcare, Buckinghamshire, UK) for later DNA extraction and SNP genotyping.

Data on demographic characteristics, clinical and gynecologic history, and use of hormone therapy for menopausal symptoms were collected at the time of recruitment.

The study protocol was approved by the local research ethics committee at *Hospital de Clínicas de Porto Alegre* (CAAE 58036616.2.0000.5327). The study was conducted in accordance with the Declaration of Helsinki, and written informed consent was obtained from each study participant at the time of recruitment.

### Measurements

All participants underwent a standardized physical examination that included measurement of blood pressure, weight, height, and waist circumference, as previously reported (19-21). Body mass index (BMI; kg/m^2^) was calculated.

The patients’ estimated 10-year ASCVD risks were calculated using the ASCVD Risk Estimator Plus (https://tools.acc.org/ascvd-risk-estimator-plus/#!/calculate/estimate/), following the guidelines recommended by the ACC. The ASCVD Risk Estimator Plus is a simple and low-cost online tool that estimates the 10-year likelihood of an ASCVD event in individuals without existing CVD, based on a set of established risk factors. The 10-year ASCVD risk score is currently categorized as low risk (<5%), borderline risk (5%-7.4%), intermediate risk (7.5%-19.9%), and high risk (≥20%). Blood pressure levels were defined according to the cutoff values recommended by the 2017 ACC/AHA guideline (22). Metabolic syndrome and the thresholds for its individual components were defined according to the Joint Scientific Statement (23).

### Laboratory analyses

Blood samples for laboratory analysis were drawn between 8 am and 10 am after a 12-hour overnight fast. Serum levels of total cholesterol, high-density lipoprotein cholesterol (HDL-c), and triglycerides (TG) were determined using colorimetric-enzymatic methods (Bayer 1800 Advia System, Mannheim, Germany), with intra-assay and interassay coefficients of variation (CVs) < 3%. Low-density lipoprotein cholesterol (LDL-c) levels were calculated using the Friedewald formula (24). Serum levels of glucose were measured using the hexokinase method (Advia 1800, Mannheim, Germany), with intra-assay and interassay CVs < 3.4%. Serum insulin levels were measured using electrochemiluminescence immunoassay (ECLIA; Roche Diagnostics, Mannheim, Germany), with a sensitivity of 0.200 μIU/mL and intra-assay and interassay CVs of 2.0% and 4.3%, respectively. Serum FSH levels were measured using ECLIA, with a sensitivity of 0.05 IU/L and intra-assay and interassay CVs of 1.8% and 3.3%, respectively. Serum estradiol levels were measured using ECLIA (Roche Diagnostics Mannheim, Germany), with a sensitivity of 5.0 pg/mL and intra-assay and interassay CVs of 5.7% and 6.4%, respectively (for statistical analysis, individual results below the test's sensitivity limit were considered to be equal to 5.0 pg/mL). Serum levels of interleukin-6 (IL-6) and tumor necrosis factor-alpha (TNF-α) were measured using multiplex immunoassay on Luminex xMAP (Merck KGaA, Darmstadt, Germany), with a sensitivity of 0.9 pg/mL and intra-assay and interassay CVs of < 2% and < 18.3%, respectively, for IL-6, and a sensitivity of 0.7 pg/mL and intra-assay and interassay CVs of < 2.6% and < 13%, respectively, for TNF-α. High-sensitivity C-reactive protein (hs-CRP) was assayed using a nephelometric method (Dade Behring Marburg, Marburg, Germany), with a sensitivity of 0.17 mg/L and intra-assay and interassay CVs of 4.4% and 5.7%, respectively.

### Genotyping

Genomic DNA samples, extracted from peripheral blood leukocytes or from FTA Elute cards following the manufacturer's DNA-specific protocol (GE Healthcare), were diluted to 2 ng/mL. To ensure the internal quality of the genotype data, 10% of blinded samples were typed in duplicate. The rs10046 C>T polymorphism in *CYP19A1* was genotyped using real-time polymerase chain reaction (RT-PCR) with allelic discrimination assays (Taqman MGB Probes), according to the manufacturer's instructions (Applied Biosystems, Foster City, CA, USA).

### Sample size calculation and statistical analysis

The sample size was calculated using data from previous studies that established an association between the CC genotype and coronary artery disease in a mixed sample of men and women (15). The WinPepi program was used to calculate the sample size, considering an 80% power and an α level of 0.05. The minimum required total sample was estimated at 266.

The Shapiro-Wilk normality test and descriptive statistics were used to assess the distribution of the data. Results are expressed as mean (standard deviation [SD]) for Gaussian variables, median (interquartile range [IQR]) for non-Gaussian variables, or percentage. Non-Gaussian variables were log-transformed for statistical analysis purposes and subsequently back-transformed into their original units of measure for reporting purposes. Group means were compared using one-way analysis of variance (ANOVA) followed by the Bonferroni *post hoc* test and Student's *t* test for independent samples. Categorical variables and the agreement of genotype frequencies with Hardy-Weinberg equilibrium were compared using the chi-square (χ²) test. Spearman's rho correlation coefficients were calculated to assess the relationships between continuous variables. Due to the small number of participants with certain cardiovascular risk factors (Table 1), the sample was grouped into tertiles of ASCVD risk for analysis, with the first, second, and third tertiles being ≤ 2.2%, 2.3%-5.5%, and ≥ 5.6%, respectively. Prevalence ratios (PRs) were estimated using univariate Poisson regression with robust variance to evaluate the relationship of the primary outcome (ASCVD risk) with age, BMI, estradiol levels, and rs10046 genotypes. The first and second tertiles of ASCVD risk were used as reference (risk < 5.6%). Variables found to be significant in the univariate model and without correlation with each other were included in the multivariate model. T carriers were grouped for analysis (CC homozygotes *versus* heterozygotes plus TT homozygotes).

**Table 1:** Anthropometric, clinical, and metabolic characteristics of 370 postmenopausal women, shown for the entire sample and stratified by rs10046 polymorphisms of the *CYP19A1* gene

Variable		All n = 370	CC n = 124	CT+TT n = 246	p
Age, years		56.07±5.58			
Time since menopause, years		6 (2-11)			
Body mass index, kg/m²		27.73 ± 5.41	27.68 ± 5.86	27.76 ± 5.18	0.887
	Eutrophic	122 (34.1%)	46 (39.0%)	76 (31.7%)	0.170^a^
	Overweight/obese	236 (65.9%)	72 (61.0%)	164 (68.3%)	
Systolic blood pressure, mmHg		128.25 ± 18.52	130.45 ± 21.05	127.13 ± 17.03	0.130
Diastolic blood pressure, mmHg		81.04 ± 11.50	82.02 ± 12.46	80.55 ± 10.98	0.248
Hypertension					
	Normal (<120 and <80 mmHg)	18.2%	17.7%	18.4%	0.885^a^
	Elevated (120-129 and <80 mmHg)	8.2%	9.7%	7.4%	
	Stage 1 (130-139 and/or 80-89 mmHg)	36.1%	34.7%	36.9%	
	Stage 2 (≥140 and/or ≥90 mmHg)	37.5%	37.9%	37.3%	
Total cholesterol, mg/dL		214.86 ± 41.91	210.57 ± 40.10	216.08 ± 41.27	0.225
HDL-c, mg/dL		56.31 ± 14.43	56.03 ± 13.95	56.45 ± 14.69	0.792
LDL-c, mg/dL		129.82 ± 33.69	127.82 ± 32.94	130.82 ± 34.09	0.425
Triglycerides, mg/dL		122 (87-166)	118 (87-159)	128.5 (87-167)	0.617
Glucose, mg/dL		91 (86-98)	91 (86-98)	91 (85-98)	0.299
IL-6, pg/mL		1.2 (0.8-2.0)	1.2 (0.8-1.8)	1.2 (0.8-2.3)	0.638
TNF-α, pg/mL		10.9 (7.1-17.6)	12.4 (7.8-17.8)	10.4 (6.7-16.9)	0.212
hs-CRP, mg/L		1.92 (0.82-4.45)	2.13 (1.02-4.63)	1.85 (0.75-4.38)	0.457
Metabolic syndrome		31.5%	31.1%	31.7%	0.917^a^
ASCVD risk		3.2 (1.7-6.7)	3.5 (1.7-6.9)	3.1 (1.8-6.4)	0.266
	<5% (low risk)	64.7%	57.7%	68.3%	0.026^a^
	5%-7.4% (borderline risk)	12.8%	18.0%	10.1%	
	7.5%-19.9% (intermediate risk)	19.8%	18.9%	20.2%	
	≥20% (high risk)	2.7%	5.4%	1.4%	
Hormone therapy		6.5%	4.8%	7.3%	0.36^a^
Diabetes		9.5%	8.9%	9.8%	0.775^a^
Use of antihypertensive drugs		36.8%	40.4%	35.0%	0.336^a^

The data are expressed as mean ± standard deviation, median (interquartile range), or percentage. P values were estimated using Student's *t* test for independent samples or Pearson's chi-square test (^a^).

Abbreviations: ASCVD, atherosclerotic cardiovascular disease; HDL-c, high-density lipoprotein cholesterol; hs-CRP, high-sensitivity C-reactive protein; IL-6, interleukin-6, LDL-c, low-density lipoprotein cholesterol; TNF-α, tumor necrosis factor-alpha.

The data were analyzed using SPSS for Windows, version 21.0 (SPSS Inc., Chicago, IL, USA). P values < 0.05 were considered significant.

## RESULTS

The clinical and metabolic characteristics of the 370 postmenopausal women included in the analysis are summarized in Table 1. The mean age was 56.07 ± 5.58 years and the mean BMI was 27.73 ± 5.41 kg/m². The median time since menopause was 6 years (2-11 years). Overall, 66% of the women were overweight or obese (BMI ≥ 25 kg/m²), 31.5% had metabolic syndrome, 9.5% had diabetes, and 36.8% were taking antihypertensive drugs. The 10-year ASCVD risk was estimated to be low, borderline, intermediate, and high in 64.7%, 12.8%, 19.8%, and 2.7% of the participants, respectively.

**Figure 1:**
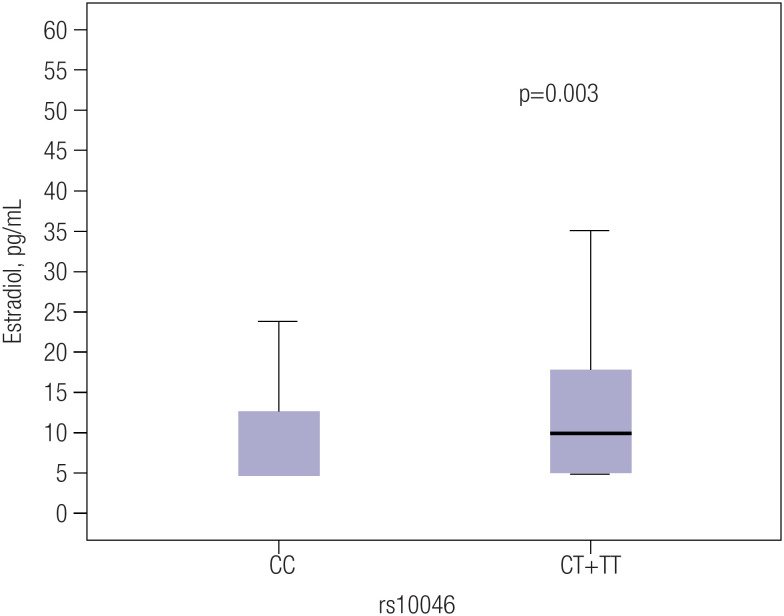
Estradiol levels according to rs10046 polymorphism genotypes. The median (interquartile range) estradiol levels in carriers of the CC and CT+TT genotypes were 5.0 pg/mL (5.0-12.6 pg/mL) and 9.8 pg/mL (5.0-17.8 pg/mL), respectively. P values were estimated using Student's *t* test for independent samples.

**Figure 2:**
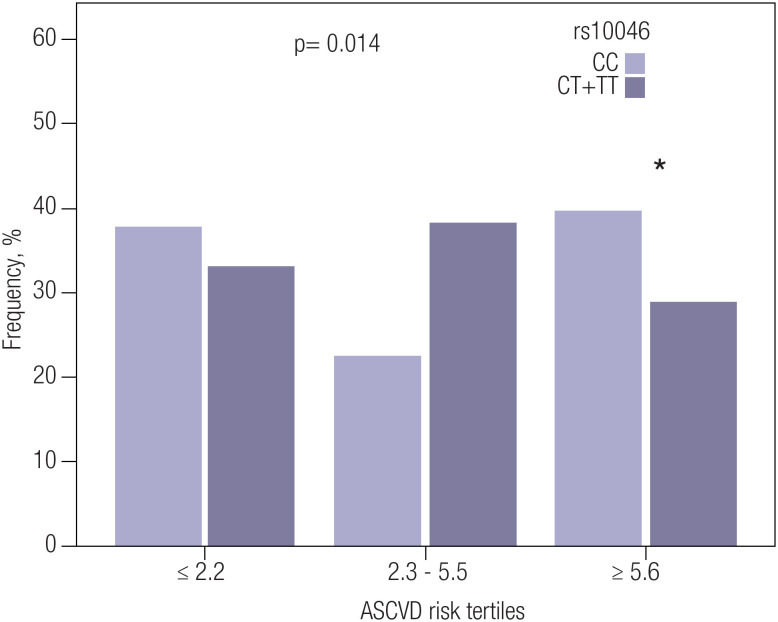
Frequency of atherosclerotic cardiovascular disease (ASCVD) risk tertiles according to rs10046 polymorphism genotypes. The frequencies of the CC and CT+TT genotypes in the highest ASCVD tertile (≥5.6%) were 39.6% and 28.9%, respectively. P value were estimated using the chi-square (χ²) test.

The rs10046 C>T polymorphism of the *CYP19A1* gene was in Hardy-Weinberg equilibrium (χ² = 1.89) with genotype frequencies of 0.33 for CC, 0.46 for CT, and 0.21 for TT, and allele frequencies of 0.56 for C and 0.44 for T. The median (IQR) estradiol levels were significantly lower in carriers of the CC genotype compared with carriers of the CT+TT genotype (5.0 pg/mL [5.0-12.6 pg/mL] *versus* 9.8 pg/mL [5.0-17.8 pg/mL], respectively, p = 0.003) (Figure 1). Also, women with a high ASCVD risk (Table 1) and within the highest ASCVD tertile (Figure 2) were more frequently carriers of the CC genotype than the CT+TT genotype (39.6% *versus* 28.9%, respectively, p = 0.014). No such association was found between the rs10046 SNP and the other isolated cardiometabolic variables (Table 1).

Table 2 presents the estradiol levels according to cardiometabolic features, showing that women on the higher tertile of ASCVD risk had lower estradiol levels compared with those in the other tertiles. A negative correlation was observed between levels of estradiol and systolic blood pressure (rho = −0.151, p = 0.004), diastolic blood pressure (rho = −0.161, p = 0.002), triglycerides (rho = −0.162, p = 0.002), and ASCVD risk (rho = −0.323, p < 0.001). No correlations were found between estradiol levels and BMI, blood pressure, glucose, or lipid categories.

**Table 2:** Estradiol levels according to cardiometabolic features

Variable		Estradiol, pg/mL	p
Body mass index, kg/m²			
	Eutrophic	7.00 (5.00-18.15)	0.251
	Overweight/obese	9.25 (5.00-16.60)	
Hypertension			
	Normal (<120 and <80 mmHg)	10.27 (5.00-18.40)	0.103
	Elevated (120-129 and <80 mmHg)	6.43 (5.00-14.63)	
	Stage 1 (130-139 and/or 80-89 mmHg)	10.20 (5.00-17.61)	
	Stage 2 (≥140 and/or ≥90 mmHg)	5.2 (5.00-15.68)	
Triglycerides			
	<150 mg/dL	9.0 (5.00-18.25)	0.062
	≥150 mg/dL	7.00 (5.00-13.30)	
HDL-c			
	>50 mg/dL	8.9 (5.00-16.31)	0.855
	≤50 mg/dL	7.0 (5.00-18.36)	
Glucose			
	<100 mg/dL	7.2 (5.00-16.92)	0.536
	≥100 mg/dL	9.5 (5.00-17.51)	
Metabolic syndrome			
	No	8.85 (5.00-17.60)	0.546
	Yes	7.00 (5.00-15.30)	
ASCVD risk tertiles			
	First (≤2.2%)	11.7 (5.81-18.96)^a^	<0.001
	Second (2.3%-5.5%)	7.0 (5.0-17.20)^a^	
	Third (≥5.6%)	5.0 (5.0-9.4)^b^	

The data are expressed as median and interquartile range. P values were estimated using Student's *t* test for independent samples or one-way analysis of variance (ANOVA). The superscript letters indicate statistical differences between groups. Abbreviations: ASCVD, atherosclerotic cardiovascular disease; HDL-c, high-density lipoprotein cholesterol.

Table 3 presents the results of regression analyses evaluating the effects of the variables of interest on the PRs for the highest tertile of ASCVD risk. In the univariate analysis, estradiol levels were significantly associated with lower PR for cardiovascular risk, while age, BMI, and the CC genotype of the rs10046 polymorphism were associated with a higher PR among women in the highest tertile of ASCVD risk (Model 1). Women with the CC genotype were 37% more likely to have an ASCVD risk ≥ 5.6%. Because age and estradiol levels clearly correlated with each other (rho = −0.314, p < 0.001) and the CC genotype was associated with lower estradiol levels (Figure 1), a multivariate Poisson regression model was constructed using age, BMI, and rs10046 polymorphism as independent variables. In this model, age and the CC genotype of rs10046 were independently associated with a higher PR for an ASCVD risk ≥ 5.6% (Model 2).

**Table 3:** Effects of age, body mass index, estradiol levels, and rs10046 polymorphism of the *CYP19A1* gene on the prevalence ratios for the highest tertile of atherosclerotic cardiovascular disease (ASCVD) risk (≥5.6%)

Variable		Model 1		Model 2	
		PR (95% CI)	p	PR (95% CI)	p
Age, years		1.172 (1.142-1.202)	<0.001	1.169 (1.140-1.200)	<0.001
Body mass index, kg/m²		1.035 (1.011-1.060)	0.005	1.019 (0.997-1.041)	0.088
Estradiol, pg/mL		0.953 (0.924-0.983)	0.002	-	-
rs10046					
	CC	1.372 (1.006-1.870)	0.046	1.352 (1.046-1.747)	0.021
	CT+TT	1	-	1	-

Model 1: univariate PRs by Poisson regression. Model 2: multivariate Poisson regression including age, BMI, and rs10046 polymorphism genotypes. Abbreviations: ASCVD, atherosclerotic cardiovascular disease; BMI, body mass index; CI, confidence interval; PR, prevalence ratio.

## DISCUSSION

In the present study, the rs10046 polymorphism in the *CYP19A1* gene was associated with lower estradiol levels and higher ASCVD scores in postmenopausal women. To the best of our knowledge, this is the first study assessing the relationships of this *CYP19A1* gene variant with the 10-year ASCVD risk assessment recommended by the ACC/AHA (3,4).

We were not able to identify any studies in the literature evaluating the rs10046 polymorphism and the ASCVD risk score in postmenopausal women, but a few studies have reported an association between this aromatase gene variant and other variables related to cardiovascular risk in different populations. A study including a Greek population with an average age of 59 years found a correlation between rs10046 and coronary artery disease in which participants with the C allele were more likely to have this disease. However, the association was not confirmed when the authors included only women in the analysis (15). A study of 1,053 Spanish women observed significant associations between the rs10046 polymorphism and both blood pressure levels and hypertension, with these associations being dependent on BMI and independent of menopausal status (16). Regarding the lipid profile, postmenopausal women with early breast cancer undergoing adjuvant aromatase inhibitor therapy who have the TT genotype have been associated with reduced triglyceride levels compared with those with the C allele (17). In Turkish adult women, the CC genotype has been associated with higher apolipoprotein B levels, independent of age or BMI (18). The same study observed that insulin levels, BMI, and the HOMA index were higher in premenopausal women who were C homozygotes than in those who carried the T allele (18).

The present study found that the CC genotype of the rs10046 polymorphism, compared with the other genotypes, occurred more frequently in women within the highest ASCVD score tertile and was associated with lower estradiol levels. The results regarding estrogen levels are in line with those of other studies. Dunning and cols. (11) found reduced estradiol levels associated with the presence of the C allele in a study of 2,115 normal postmenopausal women from the European Prospective Investigation of Cancer-Norfolk (EPIC-Norfolk) cohort. The authors also found in the same study that the C allele was associated with lower estrone levels and reduced estradiol-to-testosterone, estrone-to-androstenedione, and estradiol-to-sex hormone-binding globulin (SHBG) ratios. Similarly, analyses of data from the National Cancer Institute Breast and Prostate Cancer Cohort Consortium showed an association between the CC genotype and lower estradiol levels among premenopausal and postmenopausal women analyzed in combination (13) and among postmenopausal women alone (12), with a 12.8% increase in estradiol levels associated with the TT genotype compared with the CC genotype. Johansson and cols. (14) confirmed this association in 125 Italian postmenopausal women with estrogen-receptor-positive breast cancer.

The impact of this polymorphism on estrogen levels and its potential link to cardiovascular risk remains unclear. In the absence of a functional explanation, one hypothesis is that it is closely linked with other polymorphisms due to strong linkage disequilibrium. The (TTTA)n repeat polymorphism on the *CYP19A1* gene shows a strong correlation with the rs10046 SNP, with the longer (TTTA)n allele being significantly more common in individuals carrying the T allele of the rs10046 polymorphism (25). Published expression data suggest that higher estrogen levels are associated with a greater number of repeats in the (TTTA)n SNP; this polymorphism is also linked to the activity of the aromatase enzyme both *in vivo* and *in vitro* (26). Moreover, rs10046 is in linkage disequilibrium with rs11575899, a haplotype that has been associated with hypertension (16). Other SNPs in the *CYP19A1* gene have also been associated with cardiovascular risk (27,28) and may contribute to aromatase physiology. The rs700518 polymorphism, involving the conversion of adenine to guanine, has been associated with free estradiol index and SHBG, estradiol, and insulin levels (29), as well as with CYP19A1 expression (30). Additionally, this polymorphism has been associated with higher systolic and diastolic blood pressure levels (31). In addition to the linkage disequilibrium hypothesis, it is also worth noting that the SNP 10046 is located in the 3’UTR of the *CYP19A1* gene, which is known to be involved in the modulation of gene expression and might regulate mRNA stability by miRNA binding and/or other mechanisms. In this regard, it has been reported that the T allele of rs10046 is associated with elevated CYP19A1 mRNA levels (32,33), which can affect aromatase activity and, consequently, estrogen levels. Cardiovascular events have been reported to occur less frequently in premenopausal women than in men of the same age, but this difference disappears after menopause (34). Multiple factors, such as declining ovarian estrogen secretion and aging could largely explain the increased cardiovascular risk observed in postmenopausal women compared with premenopausal ones (6,35). This cross-talk between estradiol and cardiovascular risk is plausible since estradiol receptors are expressed in various systems, including reproductive and vascular tissues, myocardium, brain, musculoskeletal tract, and immune cells (8,36). In cardiovascular tissues, estradiol appears to regulate components of the calcium signaling machinery and numerous calcium-dependent activities through its receptors (alpha and beta receptors, and G protein-coupled estrogen receptor 1), indicating possible reciprocity between estradiol biology and calcium signaling in maintaining the system's homeostasis (37). Although the effects of menopause on hypertension are not yet fully clarified, it is possible that the reduction in arterial distensibility that occurs with advancing age is (38), at least in part, due to an increase in sympathetic activity associated with reduced estrogen levels and increased adrenergic vasoconstrictor responsiveness (39). Additionally, the hypoestrogenism following ovarian senescence contributes to endothelial dysfunction by decreasing nitric oxide bioavailability, which is secondary to reduced synthesis and increased inactivation of nitric oxide (38) through modulation of nitric oxide synthase (40).

Another possible link between estradiol and cardiovascular risk is the complex and not yet fully understood relationship between estradiol and the renin-angiotensin-aldosterone system (RAAS). The RAAS acts in many tissues (including the heart, blood vessels, adipose tissue, the adrenal gland, and the reproductive system), and experimental and epidemiological studies have shown that both circulating and tissue RAAS components are markedly affected during menopause transition, suggesting that a decrease in estradiol levels may modulate these changes (41). Indeed, beyond the well-known effects of RAAS on blood pressure levels, it acts as a pivotal immunomodulating system with vital regulatory roles throughout the body (36). The immunomodulatory function of RAAS consists of two interrelated and compensatory pathways, one proinflammatory and the other anti-inflammatory. Both pathways are essential to the regulation of cardiovascular and renal systems, influencing blood pressure and electrolyte balance. A hyperinflammatory RAAS contributes to the development of hypertension, diastolic dysfunction, heart failure, arrhythmias, left ventricular hypertrophy, and peripheral artery disease, while the proinflammatory pathway seems to offer protection against these unfavorable outcomes. Thus, in the search for a better understanding of this balance between the two immunomodulatory RAAS pathways, studies have shown that, at adequate physiological levels of estradiol, the default state of the RAAS is anti-inflammatory. Thus, thinking about the relationship between menopause and the occurrence of CVD, it is plausible to hypothesize that, with the hypoestrogenism present in the postmenopausal period, a chronic state of low-grade inflammation develops and persists in parallel with the shift of the RAAS toward its proinflammatory pathway, driving oxidative stress and cardiovascular aging (36).

Our study found an inverse correlation between estradiol and triglyceride levels and between estradiol and ASCVD risk scores. Notably, the ASCVD risk score incorporates levels of total cholesterol, LDL-c, and HDL-c into its assessment. The incidence of dyslipidemia increases throughout a woman's lifetime, and the low estrogen levels seen in the postmenopausal period are associated with proatherogenic changes in lipid profile, with increases in total cholesterol, LDL-c, apolipoproteins, and triglyceride levels, and decreases in HDL-c levels (5,42-44). While the role of estradiol in modulating lipid metabolism is more difficult to explore in humans, animal studies have highlighted some potential mechanisms. In mice, it has been shown that during the high-estradiol phase of the menstrual cycle, smaller and more efficient HDL-c particles are produced by the liver, resulting in greater cholesterol efflux from cells. This effect has been attributed to increased estrogen receptor alpha DNA binding sites when estradiol levels are high, which is speculated to promote the binding and transcriptional activity of liver X receptors, which are the main regulators of cholesterol metabolism and HDL-c efflux (45). Moreover, in murine models, high estrogen levels increase HDL-c synthesis and alter the expression of lipoprotein modifiers in liver cells (45,46). Taken together, these data reinforce the key role of estradiol in lipid metabolism in the liver.

In our study, BMI was associated with higher PRs for ASCVD risk scores. Notably, BMI is a modifiable risk factor for adverse outcomes (2,22) and lifestyle strategies for weight control have been shown to improve blood pressure levels and body composition (19). In addition, recent data from US adults aged 60-79 years (NHANES data) support the benefits of a stable weight in promoting cardiovascular health in older adults (47).

**Figure 3:**
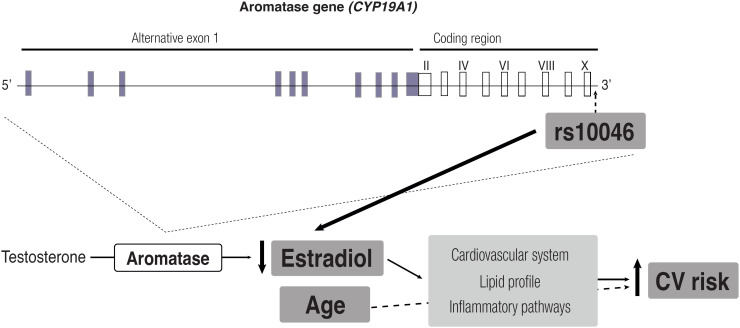
Schematic representation of the proposed contribution of the rs10046 *CYP19A1* gene variant to the increased likelihood of cardiovascular risk. The presence of the rs10046 variant of the aromatase gene (*CYP19A1*) could contribute to increased cardiovascular risk by reducing estradiol levels in postmenopausal women. Hypoestrogenism and aging are associated with adverse cardiometabolic factors, including worse lipid profiles, cardiovascular dysfunction, and inflammatory pathways.

In summary, our results suggest that the presence of the rs10046 variant of the aromatase gene (*CYP19A1*) contributes to an increased likelihood of cardiovascular risk through reduced estradiol levels. Hypoestrogenism and aging are associated with adverse cardiometabolic factors, including worse lipid profiles, cardiovascular dysfunction, and inflammatory pathways (Figure 3). The strengths of our study include the presentation of new data on Brazilian postmenopausal women, an underrepresented population in studies investigating rs10046 polymorphism and cardiovascular risk. In addition, we defined cardiovascular risk according to the ASCVD Risk Estimator Plus, following the most recent ACC/AHA recommendation, enabling the assessment of relationships between estradiol levels, rs10046 polymorphism, and varying degrees of ASCVD risk severity. The limitations of our study include its cross-sectional design, which precludes cause-and-effect analysis, and the relatively small number of participants available for comparison after stratification by specific risk factors, particularly for hypertension and ASCVD risk cutoff points. Another limitation is that we did not evaluate other polymorphisms or haplotypes, which could have provided further insight into their relationship with estradiol levels.

In conclusion, the results from the present study showed that the CC genotype of the rs10046 polymorphism of the *CYP19A1* gene is associated with low estradiol levels and increased ASCVD risk. The findings also suggest that age and the CC genotype of rs10046 are associated with a higher ASCVD risk independent of BMI among postmenopausal women. Further studies are needed to better clarify the mechanisms by which the rs10046 *CYP19A1* gene variant may be related to aromatase expression and activity in postmenopausal women.
